# Mechanism of NLRP3 inflammasome intervention for synovitis in knee osteoarthritis: A review of TCM intervention

**DOI:** 10.3389/fgene.2023.1159167

**Published:** 2023-03-29

**Authors:** Xianfu Han, Demin Lin, Weiwei Huang, Dingpeng Li, Ning Li, Xingwen Xie

**Affiliations:** ^1^ Clinical Medical College of Chinese Medicine, Gansu University of Chinese Medicine, Lanzhou, Gansu, China; ^2^ Department of Orthopedics, The Second People’s Hospital of Gansu Province, Lanzhou, Gansu, China; ^3^ Department of Orthopedics, Affiliated Hospital of Gansu University of Chinese Medicine, Lanzhou, Gansu, China

**Keywords:** NLRP3 inflammasome, synovitis, knee osteoarthritis, traditional Chinese medicine, review

## Abstract

**Objective:** This paper briefly reviews the structure and function of NLRP3 inflammasomes, signaling pathway, relationship with synovitis in KOA, and intervention of traditional Chinese medicine (TCM) in NLRP3 inflammasomes as a means to improve its therapeutic potential and clinical application.

**Method:** Literatures about NLRP3 inflammasomes and synovitis in KOA were reviewed to analyze and discuss.

**Result:** NLRP3 inflammasome can activate NF-κB mediated signal transduction, which in turn causes the expression of proinflammatory cytokines, initiates the innate immune response, and triggers synovitis in KOA. The TCM monomer/active ingredient, decoction, external ointment, and acupuncture regulating NLRP3 inflammasomes are helpful to alleviate synovitis in KOA.

**Conclusion:** The NLRP3 inflammasome plays a significant role in the pathogenesis of synovitis in KOA, TCM intervention targeting the NLRP3 inflammasome can be a novel approach and therapeutic direction for the treatment of synovitis in KOA.

## Introduction

KOA is a degenerative joint condition that is brought on by a number of reasons and frequently coexists with synovitis (Mathiessen and Conaghan, 2017). Although the pathophysiology of synovitis in KOA is not entirely clear, related research has revealed that the innate immune response plays a crucial part in the disease’s pathogenesis (Wang et al., 2018). An essential PRRs in the innate immune system, the NLRP3 inflammasome can activate the NF-κB signaling pathway by identifying pathogen-related molecular patterns (PAMPs) and damage-related molecular patterns (DAMPs), inducing an innate immune response, activating or accelerating the transmission of downstream signaling molecules, and leading to synovitis (Huang et al., 2022). In order to provide a theoretical foundation and point of reference for the diagnosis and treatment of synovitis in KOA, this article reviews and analyzes historical data regarding the role of the NLRP3 inflammasome for synovitis in KOA as well as the research status of TCM interventions on the NLRP3 inflammasome.

## The structure and function of NLRP3 inflammasome

The inflammasome is a multiple proteins complex that exists in the cytoplasm of cells. It was first proposed by Martinon et al. (2002). It is mainly formed during the activation of caspase-1 by nucleotide-binding oligomerization domain (NOD) like receptors in PRRs. NOD-like receptors play an important role in innate immunity, among which NLRP3 inflammasome is the most deeply studied (Zhang et al., 2021a). NLRP3 consists of an amino-terminal pyridine domain (PYD), a central NACHT domain, and a carboxyl-terminal leucine-rich repeat (LRR) (Gaul et al., 2021). Studies have shown that the NACHT domain has ATP binding activity to promote the oligomerization of NLRP3, LRR and NACHT domains form a mutual inhibitory effect, and the PYD domain allows NLRP3 to interact with other inflammasome proteins (Hafner-Bratkovič et al., 2018). NLRP3 exists in the cytoplasm and participates in innate immunity as PRRs. It is activated by recognizing PAMPs and DAMPs (Zhao et al., 2022a). NLRP3 inflammasome consists of NLRP3 (nucleotide-binding domain leucine-rich repeat (NLR) and pyrin domain containing receptor 3), ASC (apoptosis-associated speck-like protein containing a caspase recruitment domain), and Pro-caspase-1 (Zahid et al., 2019). NLRP3 is considered to be the site of the sensing activation signals. ASC is the adaptor protein of NLRP3 inflammasome, which connects NLRP3 and Pro-caspase-1. The phosphorylation of ASC promotes the activation of the inflammasome. Pro-caspase-1 has no catalytic activity, but it can be activated into the effector protein Caspase-1 of the NLRP3 inflammasome by its shearing. Caspase-1 can transform inactive Pro-IL-1β and Pro-IL-18 into mature IL-1β and IL-18 (Huang et al., 2021). It has been found that NLRP3 is easily activated in dendritic cells, macrophages, and neutrophils (Zhao et al., 2022b). The NLRP3 inflammasome pathway belongs to the classical inflammasome pyroptosis pathway (Caspase-1 mediated). In addition, there are non-classical inflammasome pyroptosis pathways (Caspase-4, Caspase-5, Caspase-11 mediated) and apoptotic protein Caspase-3 mediated pyroptosis pathway (Moretti et al., 2022; Fu et al., 2021; Zhang et al., 2021b). The role of NLRP3 inflammasome for synovitis in KOA is a hot topic in recent years, many studies have shown that NLRP3 inflammasome is a potential mechanism of synovitis in KOA, but it needs to be further studied.

## NLRP3 inflammasome signaling pathway

NLRP3 inflammasome mainly senses stimulation signals in cells and can be activated by a variety of internal and external factors, such as PAMPs and DAMPs, including lipopolysaccharide (LPS), amyloid β, cholesterol crystals, monosodium urate crystals (MSU), adenosine triphosphate (ATP), fatty acids, and hyaluronic acid. Some bacteria and fungi can also activate NLRP3 as PAMPs. In addition to the above factors, crystal or granular structures such as silica, asbestos, and alum can also activate NLRP3 and cause inflammatory cascade amplification (Kelley et al., 2019; Swanson et al., 2019; McGettrick et al., 2020).

**FIGURE 1:**
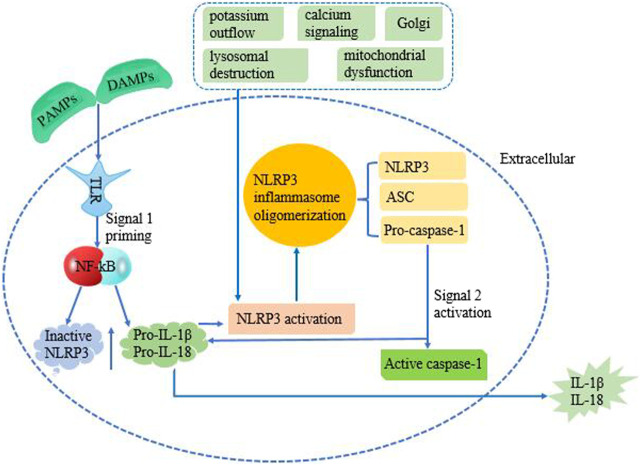
Mechanism of NLRP3 activation requires two signals. The first priming signal is provided through the interaction of PAMPS/DAMPs with TLRs. This initiates NF-κB signaling, which upregulates the production of pro-IL-1β, pro-IL-18, and inactive NLRP3 protein. The second step is an activation signal which causes NLRP3, ASC, and Pro-caspase-1 to come together. Pro-caspase-1 is then converted into active caspase-1, along with NLRP3 and ASC forms the NLRP3 inflammasome complex. Active caspase-1 cleaves pro-IL-1β and pro-IL-18 causing their activation, which converts into IL-1β and IL-18, subsequent release to extracellular. The molecular mechanisms of NLRP3 inflammasome activation mainly include potassium outflow, calcium signaling, lysosomal destruction, mitochondrial dysfunction, and Golgi.

It has been found that there are two signal models for NLRP3 inflammasome activation (Figure 1): the first step is initiated at the transcriptional level, in which Toll-like receptors recognize PAMPs or DAMPs to activate NF-κB-mediated signal pathway, which increases the production of pro-IL-1β, pro-IL-18 and NLRP3 proteins. The second step is the activation signal, which initiates NLRP3 oligomerization and causes NLRP3, ASC, and Pro-caspase-1 to form inflammasomes. Subsequently, Pro-caspase-1 is self-sheared and activated to Caspase-1 p10 and Caspase-1 p20. After Caspase-1 is activated, pro-IL-1β and pro-IL-18 can be sheared into mature IL-1β and IL-18 (Li et al., 2021; Mu et al., 2022; Zhang et al., 2022; Zhang et al., 2018; Pei et al., 2022). Then released to the outside of the cell, and more inflammatory cells (HMGB1, leukotrienes, prostaglandins, etc.) were collected, which led to the cascade of inflammation.

The molecular mechanisms of NLRP3 inflammasome activation mainly include potassium outflow, calcium signaling, lysosomal destruction, mitochondrial dysfunction, and Golgi. Potassium ion outflow causes a decrease in intracellular potassium levels under the stimulation of ATP, pore-forming toxins, crystals, particles, etc. Then directly binds and activates NLRP3 under the action of NIMA-associated kinase 7 (Nek7) (Sun et al., 2022). Plant-derived dietary lectins are internalized, then escaped from the lysosome and are transported to the endoplasmic reticulum. Endoplasmic reticulum-loaded lectins trigger calcium ion release and mitochondrial damage. It was found that blocking the flow of calcium ions can inhibit NLRP3 inflammasome components and activation. Promoting calcium ion release can aggravate mitochondrial damage, and mediated mitochondrial damage can cause NLRP3 inflammasome activation. And promoting the release of calcium ions can aggravate the injury of mitochondria, and calcium ion-mediated mitochondrial damage could cause the activation of NLRP3 inflammasome (Murakami et al., 2012). Lysosomal damage releases cathepsin B directly binds to the NLRP3 inflammasome and promotes the activation of the NLRP3 inflammasome (Ma et al., 2022). The release of mitochondrial ROS (mt ROS) and mitochondrial DNA (mt DNA) caused by mitochondrial dysfunction is another important cause of NLRP3 inflammasome activation. For example, after the increase of ROS caused by NLRP3 agonist, the redox stress mediated by thioredoxin interacting protein (TXNIP) can activate the NLRP3 inflammasome (Luo et al., 2022). It was found that the Golgi apparatus is involved in NLRP3 inflammasome activation through protein kinase D signaling on mitochondria-associated endoplasmic reticulum membranes (Zhang et al., 2017). In addition, some infectious microorganisms have been shown to activate the NLRP3 inflammasome (Giraud et al., 2019). In conclusion, NLRP3 inflammasome is a key host immune defense mechanism for the body to face PAMPs or DAMPs. With the deepening of research, NLRP3 inflammasome will provide more ideas for the treatment of many diseases.

## The role of NLRP3 inflammasome for synovitis in KOA

### The expression of NLRP3 inflammasome in KOA synovium

Synovitis is one of the important causes of cartilage degeneration (Oka et al., 2021). IL-1β involved in cartilage degradation may be produced by synovial cells rather than chondrocytes (Wang et al., 2021). Synovitis is relatively more studied in rheumatoid arthritis (RA). It has been found that NLRP3 inflammasome is highly activated in the synovium of RA patients and collagen-induced arthritis mice. The activation of NLRP3 inflammasome mainly occurs in infiltrating monocytes/macrophages in the synovium. The NLRP3 inhibitor MCC950 can significantly inhibit the activation of NLRP3 inflammasome in the synovium and reduce the production of IL-1β (Guo et al., 2018). Clavijo-Cornejo et al. found that the protein expression of NLRP3 in the synovium of KOA patients increased 5.4-fold with respect to normal patients (Clavijo-Cornejo et al., 2016). Sakalyte et al. found that NLRP3 inflammasome existed in synovial fibroblast cell of KOA patients and showed high expression (Sakalyte et al., 2022). The activation of NLRP3 inflammasome promotes synovitis, which participates in the whole process of KOA and promotes the progress of KOA.

### NLRP3 inflammasome mediates synovitis in KOA

The course of synovitis in KOA often involves the participation of immune cells, and innate immunity is an important barrier for the human body to prevent the invasion of pathogens. PRRs can recognize and perceive DAMPs or PAMPs, and combine with them to form ligand polymer, which can cause and promote synovitis in KOA after activating the innate immune response (Leung et al., 2015). NLRP3 inflammasome, as a PRRs, can activate the NF-κB signal pathway after combining with DAMPs and PAMPs expressed or secreted in the synovium, causing the expression of pro-inflammatory cytokines and inflammatory mediators, then leading to synovitis. Which can promote synovial cell proliferation, and aggravate synovitis (Zhang et al., 2019a). In KOA synovial macrophages, NLRP3 inflammasomes are induced and released into the synovial fluid and surrounding tissues under the action of different DAMPs. Which increased the expression levels of IL-1β and IL-18 in a series of inflammatory reactions involving synovial macrophages and chondrocytes (An et al., 2020). Eventually, this led to synovitis and cartilage degeneration.

Chen et al. found that the Nrf2/HO-1 signal in the synovium of KOA patients and model rats may be an important way to activate the NLRP3 inflammasome. Oxidative stress induced by ROS may be the main reason for the activation of NLRP3 inflammasome and the subsequent release of downstream pro-inflammatory factors in the development of KOA (Chen et al., 2019). The activation of NLRP3 inflammasome can induce the secretion of proinflammatory cytokines IL-1β and IL-18, leading to the aggravation of downstream inflammatory response and accelerating the occurrence of synovitis in KOA. In addition to ROS, the ectopic deposition of hydroxyapatite (HA) crystals in joints are related to the pathogenesis of synovitis in KOA. HA crystals induce macrophages to secrete IL-1 and IL-18 in an NLRP3 inflammasome-dependent manner. In addition, calcium crystals in the synovial fluid of KOA patients showed NLRP3 inflammasome stimulating activity *in vitro* (Jin et al., 2011). It was found that the level of uric acid was positively correlated with the expression of IL-18 and IL-1β in synovial fluid of KOA patients, while uric acid could activate NLRP3 inflammasome and increase the expression of IL-18 and IL-1β, then led to the aggravation of synovitis. This indicates that there was a close relationship between NLRP3, uric acid, and proinflammatory cytokines (Aibibula et al., 2016). HA crystal, MSU crystal, calcium pyrophosphate, and calcium phosphate also were inflammasome activators (Busso and So, 2012). Zhao et al. found that NLRP3 inflammasome in the synovium of KOA patients was involved in synovial fibroblast cell inflammation and pyroptosis. Inhibition of NLRP3 inflammasome can significantly reduce the expression of apoptosis-related cytokines (Zhao et al., 2018). Xiao et al. found that NLRP3 inflammasome mediated synovial fibroblast cell pyroptosis can enhance the secretion of high mobility group protein B1 (HMGB1), and HMGB1 has a pro-inflammatory effect and aggravates synovitis (Xiao et al., 2021). Zhang et al. found that hypoxia in the synovium of KOA model rats led to an increase in hypoxia-inducible factor 1α (HIF-1α), resulting in an increase in the expression of NLRP3, Caspase-1and GSDMD. Thereby aggravating synovitis and fibrosis in KOA (Zhang et al., 2019b).

## TCM interventions on the NLRP3 inflammasome for synovitis in KOA

The intervention effect of TCM on the NLRP3 inflammasome for synovitis in KOA *via* TCM monomer/active ingredient, Decoction, External ointment, and Acupuncture (Table 1).

**TABLE 1:** Traditional Chinese Medicine against synovitis in KOA.

Species	Drugs/methods	Research object	Mechanism
TCM Monomer/Active ingredient	Casticin	Rats/FLS	Inhibits the activation of HIF-1α/NLRP3 inflammasome
	Agnuside	Rats/FLS	Inhibits the activation of HIF-1α/NLRP3 inflammasome
	Chrysin	Rats	Inhibits the activation of NLRP3 inflammasome
	Vanillic Acid	Rats/FLS	Decreases the expression of caspase-1, ASC, and NLRP3 and also reduce the levels of IL-1β and IL-18
	Nodakenin	Mice/Chondrocytes	Regulates the mitochondrial Drp1/ROS/NLRP3 axis
	Isochlorogenic acid A	Rats	Decreases the activation of NLRP3 inflammasome and NF-κB phosphorylation expression
	Xanthotoxol	Rats	Inhibits the infiltration of inflammatory factors and downregulates the activity of the NF-κB signal pathway by inhibiting the activation of NLRP3 inflammasome
	Andrographolide	Mice/Chondrocytes	Regulates the circ_Rapgef1/miR-383–3p/NLRP3 signaling axis
Decoction	Xibining	Rats	Inhibits the activation of HIF-1α/NLRP3 inflammasome
	Du Huo Ji Sheng Tang	Human/Rat	Suppresses NLRP3/NF-κB inflammatory signals
External Ointment	Layers Adjusting External Application	Rats	Suppresses the expression of NLRP3, ASC, Caspase-1 protein and mRNA in the synovium
	“Sanse Powder” Essential Oils Nanoemulsion	SD rats/FLS	Inhibits the ERS/TXNIP/NLRP3 signaling axis
	“Sanse Powder” Volatile Oil	FLS	Inhibits the activation of NLRP3 inflammasome
Acupuncture	electroacupuncture	SD rats	Inhibits the NLRP3 inflammasome signaling pathway and reducing pyroptosis
	electroacupuncture	Guinea pigs	Suppresses the activation of NLRP3 inflammasome
	moxibustion combined with ultrashort wave	Human	Suppresses NLRP3 inflammasome signaling pathway

### TCM monomer/active ingredient

#### Casticin

Casticin is a compound purified from the TCM Viticis Fructus. In rats KOA model induced by monoiodoacetic acid (MIA) and the inflammation of primary FLS stimulated by lipopolysaccharide (LPS), Casticin can improve hypoxia, inflammation of synovium and synovium fibrosis in rats. In addition, Casticin can inhibit the activation of NLRP3 inflammasome in rats KOA model and FLS, indicating that Casticin alleviates MIA-induced synovitis in KOA by inhibiting the activation of HIF-1α/NLRP3 inflammasome (Li et al., 2020a).

#### Agnuside

Agnuside is a non-toxic natural small molecule isolated from the extract of Vitex negundo. In MIA-induced rats KOA model and LPS-induced FLS inflammation model, it was found that Agnuside could effectively alleviate local hypoxia in the synovium, reduce the mRNA and protein levels of HIF-1α, caspase-1, ASC, and NLRP3. Meantime downregulate the expression of NLRP3 inflammasome downstream factors IL-1β and IL-18, also fibrosis markers TGF-β, TIMP1, and VEGF. It is indicated that Agnuside reduces synovitis and fibrosis in experimental KOA by inhibiting the activation of HIF-1α/NLRP3 inflammasome (Zhang et al., 2021c).

#### Chrysin

Chrysin is a natural flavonoid found in Scutellaria baicalensis Georgi. In the rats KOA model induced by MIA, Chrysin can not only reduce synovitis but also reduce the secretion of pain-related factors, and increase the mechanical pain threshold and cold pain threshold of rat. Chrysin alleviates synovitis by inhibiting NLRP3 inflammasome activation and IL-1β expression. It is suggested that Chrysin can reduce synovitis in KOA and improve pain behavior in rats, which may be related to the ability to inhibit the activation of NLRP3 inflammasome (Liao et al., 2020).

#### Vanillic acid

Vanillic Acid is a monomer from Chinese herbal medicine. It was found that Vanillic acid decreased the expression of caspase-1, ASC, and NLRP3 in rats KOA model both *in vivo* and vitro and also reduced the levels of IL-1β and IL-18, which reduced synovium fibrosis and alleviated pain-related behaviors in rats KOA model. The expression of pain mediators CGRP, NGF, and TrkA in FLS was downregulated. It shows that Vanillic Acid reduces synovitis and pain-related behaviors in rats KOA model (Ma et al., 2021).

#### Nodakenin

Nodakenin is the main coumarin active ingredient in Angelicae Pubescentis Radix. It was found that Nodakenin could increase trabecular bone score in subchondral bone, reduce the level of serum inflammatory factors and alleviate synovitis in mice KOA model after Nodakenin intervention. *In vitro*, it was found that Nodakenin inhibited the phosphorylation of kinesin-related protein 1 (Drp1) and ROS production in chondrocytes stimulated by LPS through DRP1-dependent mitochondrial division. In addition, Nodakenin inhibited the mRNA levels of inflammatory factors (COX 2, IL-1β, and TNF-α), NLRP3 inflammasome, and MMP13 in activated chondrocytes. It indicated that Nodakenin alleviates cartilage degradation and synovitis in KOA by regulating the mitochondrial Drp1/ROS/NLRP3 axis (Yi et al., 2022).

#### Isochlorogenic acid A

Isochlorogenic acid A, as a natural product of quinic acid and caffeic acid by esterification and condensation, mostly exists in *Lonicera japonica*, Celastrus angulatus, *L. japonica*, and other plants. Isochlorogenic acid A can significantly reduce the expression of NLRP3, caspase-1, NF-κB p65, p-NF-κB p65, p-IκB, and RANKL in the synovium of collagen-induced arthritis rats, downregulate plasma IL-1β, IL-6, TNF-ɑ, CRP, IFN-γ and IL-18, and reduce the swelling of rats toes. Isochlorogenic acid A has a good anti-inflammatory effect on collagen-induced arthritis, and its anti-inflammatory activity may be related to decreasing the activation of NLRP3 inflammasome and NF-κB phosphorylation expression (Liu et al., 2019).

#### Xanthotoxol

Xanthotoxol is a coumarin compound extracted from Chinese herbal medicine’s common cnidium fruit. In the rats KOA model established by papain, xanthotoxol can significantly reduce joint swelling, synovial hyperemia, and synoviocyte proliferation, meantime reduce synovium inflammatory cell infiltration and vascular proliferation.

It can significantly reduce the levels of IL-6, IL-1β, and TNF-α in synovial fluid, and reduce the content of NLRP3 protein and NF-κB phosphorylated protein in synovium. Xanthotoxol inhibits the infiltration of inflammatory factors and downregulates the activity of the NF-κB signal pathway by inhibiting the activation of NLRP3 inflammasome. Thereby inhibiting the expression of inflammatory factors, relieving synovitis in KOA, and exerting a protective effect on osteoarthritis (Zhuang et al., 2019).

#### Andrographolide

Andrographolide is the main active ingredient of the natural plant Andrographis paniculata. Andrographolide can reduce the infiltration of inflammatory cells in synovium, and inhibit the inflammatory response in mice KOA model established by anterior cruciate ligament transection (ACLT). It can inhibit the proliferation, apoptosis, and inflammation of chondrocytes induced by LPS stimulation. Andrographolide inhibits the progression of osteoarthritis by regulating the circ_Rapgef1/miR-383–3p/NLRP3 signaling axis (Yan et al., 2022).

### Decoction of TCM

#### Xibining

Xibining (patent number: CN201010514325) is a TCM compound developed by Professor Peimin Wang aiming at KOA clinical treatment with the therapeutical principle of warming channels and activating blood circulation. Medicine composition and dosage: Radix Aconiti Carmichael 15 g, Processed cibotium barometz 15 g, human placenta 10 g, Cornus officinalis 1 5g, Wilson cinnamon bark 15 g, Morinda officinalis 10 g, Jobstears seed 10 g, Tuber fleece flower root 10 g, Medicinal cyathia root 10 g, Radix glycyrrhiza 5 g. In the rats KOA model established by sodium iodoacetate, after xibining treatment, the infiltration of inflammatory cells in the synovium of rats KOA decreased. The infiltration of inflammatory cells, mRNA, and protein expression of HIF-1α, NLRP3, ASC, GSDMD, and Caspase-1 in synovium were decreased. Meantime the levels of IL-1β and IL-18 in synovium decreased. Thereby Xibining can effectively improve the hypoxia condition of the synovium in KOA, reduce the expression of HIF-1α, reduce the activation of the NLRP3 inflammasome, and reduce synovitis in KOA (Zhang et al., 2020).

#### Du Huo Ji Sheng Tang

Du Huo Ji Sheng Tang (DHJST) is a TCM formula, which is a classic prescription for the treatment of KOA. The levels of serum IL-1β, IL-6, IL-10, TNF-α, NLRP3, ASC, Caspase-1, p-NF-κB-P65, and p-IκBa were decreased in KOA patients after DHJST treatment. In the rats KOA model established by Papain Enzyme, after DHJST intervention, the swelling volume of the right hind foot of the rats was significantly reduced, and the levels of IL-1β, IL-6, and TNF-α in synovial fluid of the knee joint were downregulated, meantime the expression of NLRP3, ASC, Caspase-1, p-NF-κB-P65, and p-IκBa in the synovium of the knee joint was decreased, and the pathological changes such as synovitis and cartilage degeneration of the knee joint were alleviated. DHJST alleviated KOA by suppressing NLRP3/NF-κB inflammatory signals in rats (Chen et al., 2020).

## External ointment of TCM

### Layers Adjusting External Application

Layers Adjusting External Application (Patent No: ZL200820185241.8) is a TCM ointment for external use, which is composed of Chinese medicines for warming meridians and activating blood circulation. Layers Adjusting External Application can improve the Krenn score of synovitis in rats KOA model, downregulate the expression of serum IL-1β and TNF-α, downregulate the expression of NLRP3, ASC, Caspase-1 protein and mRNA in the synovium, meantime downregulate levels of MMP-1 and MMP-13 in cartilage. Layers Adjusting External Application may inhibit synovitis in KOA by down-regulating the expression of NLRP3 and Caspase-1, reducing the level of cartilage MMPs, and playing a role in protecting cartilage (Li et al., 2020b).

“Sanse Powder”

“Sanse Powder” is the core component of Layers Adjusting External Application (Patent No: ZL200820185241.8). It is a hospital preparation of the Department of Orthopedics and Traumatology of the Affiliated Hospital of Nanjing University of Traditional Chinese Medicine. It is one of the representative prescriptions for warming meridians and activating blood circulation. In rats synovitis in KOA model and FLS stimulated by LPS, “Sanse Powder” Essential Oils Nanoemulsion can inhibite ERS/TXNIP/NLRP3 signaling axis to regulate the excessive production of IL-1β and IL-18 (Liu et al., 2021). In KOA inflammatory cell model established by LPS, “Sanse Powder” Volatile Oil can downregulate the protein and mRNA expression of NLRP3, caspase-1, and ASC, meantime reduce the levels of IL-1β and IL-18 in cell supernatant. It may play a role in improving synovitis in KOA by inhibiting the activation of NLRP3 inflammasome in FLS and reducing the downstream inflammatory cascade (Liao et al., 2021).

### Acupuncture

In the KOA model of SD rats established by Papain Enzyme, after electroacupuncture stimulation of “Neixiyan” (EX-LE4) and “Dubi” (ST35), the pathological score of synovium, serum IL-1β, and IL-18 levels, synovium NLRP3, ASC, Caspase-1, IL-1β, IL-18 mRNA and protein expression levels were decreased, meantime the expression of GSDMD mRNA and GSDMD-N protein was also decreased. Electroacupuncture can reduce the inflammatory response of knee joint synovium in rats, which may be related to inhibiting the NLRP3 inflammasome signaling pathway and reducing pyroptosis (Yu et al., 2022). In the guinea pigs KOA model, after electroacupuncture treatment, the mechanical withdrawal threshold of guinea pigs was downregulated, the articular cartilage structure was improved, and the fibrosis on the cartilage surface was reduced. Electroacupuncture can inhibit the activation of the NLRP3 inflammasome, and inhibit the protein expression levels of caspase-1 and IL-1β in cartilage tissue. Electroacupuncture alleviates KOA pain by suppressing the activation of NLRP3 inflammasome (Wang et al., 2021). Wang et al. observed the effect of moxibustion combined with ultrashort wave on elderly patients with KOA. The results showed that the total effective rate of the observation group was 90.48%. After treatment, the VAS and WOMAC scores of the observation group decreased, and the Lysholm knee joint scores increased. The serum IL-1β, TNF-α, SOD, MDA, miR-155, and NLRP3 were all lower than those before treatment. The results show that moxibustion combined with an ultrashort wave can effectively improve the knee joint pain and function of elderly KOA patients, reduce oxidative stress response, and the potential mechanism may be through Suppressing NLRP3 inflammasome signaling pathway (Wang et al., 2022b).

## Conclusion

The NLRP3 inflammasome plays a significant role in the pathogenesis of synovitis in KOA, and innate immunity is activated during the pathogenesis of this condition. The NF-κB signaling pathway, pro-inflammatory factor production, inflammatory mediator secretion, synovitis in KOA, and synovial cell proliferation can all be brought on by the activation of the NLRP3 inflammasome. The pathophysiology of synovitis in KOA can be further understood by analysis of the role of the NLRP3 inflammasome. Targeting the NLRP3 inflammasome can be a novel approach and therapeutic direction for the treatment of synovitis in KOA. The research conclusions are mostly from animal or *in vitro* experiments. The effectiveness and safety of clinical applications are not completely clear. Further depth research is needed.
